# N-Acetyl-Cysteine-Loaded Biomimetic Nanofibrous Scaffold for Osteogenesis of Induced-Pluripotent-Stem-Cell-Derived Mesenchymal Stem Cells and Bone Regeneration

**DOI:** 10.3389/fbioe.2021.767641

**Published:** 2021-12-15

**Authors:** Xiaolei Li, Feng Xiong, Shuguang Wang, Zhuojun Zhang, Jihang Dai, Hui Chen, Jingcheng Wang, Qiang Wang, Huihua Yuan

**Affiliations:** ^1^ Department of Orthopedics and Orthopedic Institute, Clinical Medical College of Yangzhou University, Subei People’s Hospital of Jiangsu Province, Yangzhou, China; ^2^ School of Life Sciences, Nantong University, Nantong, China

**Keywords:** bone tissue engineering, n-acetyl cysteine, HAp/SF nanofibers, drug release, osteogenesis

## Abstract

To regenerate bone tissues, we investigated the osteogenic differentiation of induced-pluripotent-stem-cell-derived mesenchymal stem cells (iPSC-MSCs) and bone regeneration capacities using N-acetyl cysteine (NAC)-loaded biomimetic nanofibers of hydroxyapatite/silk fibroin (HAp/SF). The addition of HAp and NAC decreased the diameters of the electrospun fibers and enhanced the mechanical properties of the silk scaffold. The release kinetic curve indicated that NAC was released from NAC/HAp/SF nanofibers in a biphasic pattern, with an initial burst release stage and a later sustained release stage. This pattern of release of NAC encapsulated on the NAC/HAp/SF scaffolds prolonged the release of high concentrations of NAC, thereby largely affecting the osteogenic differentiation of iPSC-MSCs and bone regeneration. Thus, a new silk electrospun scaffold was developed. HAp was used as a separate nanocarrier for recharging the NAC concentration, which demonstrated the promising potential for the use of NAC/HAp/SF for bone tissue engineering.

## Introduction

The combination of a pure bone tissue engineering scaffold and seed cells can repair damaged bone tissue to a certain extent. However, it cannot provide close and effective information connection with surrounding natural organs and tissues to accelerate healing of bone tissue damage. The functional bionics of bone tissue engineering scaffolds are based on this signal factor, one of the three elements of bone tissue engineering (Collignon et al., 2017). Bone tissue can produce growth factors during repair (including bone morphogenetic protein-2 (Groeneveld and Burger, 2000), insulin-like growth factor (Koch et al., 2005), basic fibroblast growth factor (Du et al., 2012), transforming growth factor-β (Hong et al., 2000), and vascular endothelial growth factor (Mayr-Wohlfart et al., 2002)). Multiple growth factors coordinately control the behavior of bone cells and accelerate the secretion of extracellular matrix in osteoblasts. Bone tissue engineering can also induce osteoblast proliferation and promote bone regeneration after separation and purification of these growth factors.

The most frequently used functional bionics in bone tissue engineering is to select the growth factor that participates in bone repair as a biological activity factor to prepare the bone scaffold and to accelerate bone repair by regulating the rate of release of the growth factor. Functional bionics is also referred to as a third generation of bioactive composite material and biological hybrid material, a compound material, combined with the biological activity of cytokines. It can actively stimulate and induce the self-repair and regeneration of the injured tissue. Thus, damaged tissue and organs can eventually be replaced by healthy tissues or organs (Boccaccini and Blaker, 2005). Growth factors cannot be widely used in clinics because of the difficulties in extraction, high price, susceptibility to inactivation, short half-life, and immunogenicity to body tissue (James et al., 2016). Small-molecule drugs have good efficacies and stable pesticide effects. They do not easily become inactive during the preparation of scaffolds and have low molecular weights, high solubilities, and diverse structures and functions. They can also be designed and improved according to the requirements and easily produced in large quantities. They attracted considerable attention because of their known molecular structure, low cost, and simple commercialization (Banaszynski et al., 2006; Ontoria et al., 2009). Accordingly, the use of small-molecule drugs with good properties, instead of growth factors, has become a trend in bone tissue engineering (Xu et al., 2008).

N-acetyl cysteine (NAC), a water-soluble and membrane-permeable small molecule, has various bio-functionalities, including antioxidant activity, ability to improve cytocompatibility, and osteogenic differentiation (Oikawa et al., 1999; Yamada et al., 2013; Zhu et al., 2015). NAC loaded on a collagen sponge scaffold promotes bone regeneration *in vitro* and *in vivo* (Yamada et al., 2013). However, NAC is simply absorbed onto this scaffold, which may result in a burst of release of NAC thereby imposing limitations in biological and clinical applications. Therefore, the enhancement of bone regeneration relies on the effective incorporation and viable release of bioactive molecules in a controlled manner over a comparatively long period of bone regeneration. Recently, an NAC-loaded polylactic-co-glycolic acid electrospun system with mesoporous silica nanoparticle nanocarriers was developed for the promotion of osteogenesis of rBMSCs *in vitro* (Zhu et al., 2019). Although these systems can achieve controlled delivery of NACs *in vitro*, in the clinical settings of bone tissue repair and regeneration, scaffolds as bio-mimetics of the natural bone composition of hydroxyapatite (HAp) and collagen nanofibers are required (Sell et al., 2007). Thus, the fabrication of composite nanofibers consisting of HAp is a rational strategy. HAp-incorporated silk fibroin (HAp/SF) has been considered one of the most attractive biomaterial scaffolding systems for bone tissue engineering (Farokhi et al., 2018).

In this study, HAp/SF composite nanofibers were used as carriers to control the release of NAC and investigate its effect on fiber properties. The effects of HAp/SF composite nanofibers loaded with NAC on the osteogenic differentiation of induced-pluripotent-stem-cell-derived mesenchymal stem cells (iPSC-MSCs) were evaluated at the cellular, protein, and genetic levels. We created a mouse cranial bone defect model and assessed the efficacy of the NAC/HAp/SF nanofibrous scaffold for bone regeneration. This nanofibrous scaffold could maximize the osteogenic ability of iPSC-MSCs in the long term and could be used for personalized and functional bone repair and regeneration applications.

## Materials and Methods

### Preparation of NAC/Hydroxyapatite/Silk Fibroin Nanofibers

HAp/SF composite fibers with 10% HAp were selected as drug carriers to prepare drug-loaded fibers with a certain amount of NAC. The blending method was used to disperse NAC evenly in the spinning solution. Drug-loaded fibers were prepared by electrospinning. A solution was prepared by dissolving the required weight of NAC in 10 μl of water and adding it to the spinning solution, followed by stirring, and mixing. 0.4 g of HAp was added to a stirring bottle containing 2 ml of methane acid. The HAp particles were evenly dispersed by ultrasonication for 30 min 0.4 g of SF and 0.0082 g of polyethylene oxide (PEO) were then added to the solution and stirred and dissolved. After stirring for 1 h, 10 μL of an aqueous solution containing NAC (0.78 mg) was added, and the NAC/HAp/SF solution was then prepared by magnetic stirring for 5 h at room temperature. The electrospinning was carried out at a spinning voltage of 7–8 kV, an injection rate of 0.4 ml/h, a receiving distance of 15 cm, at room temperature, and an ambient humidity of 30–40%.

### Characterization of N-Acetyl Cysteine/Hydroxyapatite/Silk Fibroin Nanofibers

#### Morphology of Nanofibers

Morphologies of the different NAC/HAp/SF drug-loaded fiber membranes were observed by scanning electron microscopy (SEM, ZEISS Gemini SEM 300, Germany) and transmission electron microscopy (TEM, Talos F200X, FEI, United States). After a double-sided conductive adhesive was attached to the SEM sample stage, the different electrospun fiber membranes (after evaporation of the solvent) were cut into appropriate sizes and attached to the conductive adhesive. The sample was then sprayed with gold for 40 s under vacuum. Finally, the surface morphology of the electrospun fiber membrane was observed using SEM at an accelerating voltage of 10 kV and imaged as needed. The diameter of the fiber was measured using the ImageJ software (at least 50 times per sample).

### Hydrophilic Performance Test

The contact angles of the different drug-loaded fiber membranes were measured using a contact-angle tester (JCY-1, Shanghai Fangrui Instrument Co., Ltd.). 0.3 μL of deionized water was dropped on the sample. The morphologies of the water droplets on the fiber membrane at different times were imaged. The angle between the water droplet and fiber membrane was measured to obtain the contact angle.

### Test of Drug Release

Ultraviolet spectrophotometry (Thermo Scientific Evolution 300, United States) was used to analyze drug release. Scaffold samples (20 mg, alcohol gas treated for 1 h) were immersed in 2 ml of distilled deionized water. The samples were then mixed with a 4-chloro-7-nitrobenzofurazan (0.006 wt%) chromogenic agent in a ratio of 1:18. After 30 min of reaction, the NAC concentrations were calculated based on the absorbance at 423 nm.

### *In-vitro* Cyto-Compatibility of the N-Acetyl Cysteine /Hydroxyapatite/Silk Fibroin Nanofibers

The rat iPSC-MSCs was induced by the rat iPS cells (Sidansai Biotechnology, Shanghai, China, China) cultured in MSCs medium and consisted of Dulbecco’s Modified Eagle’s Medium (Hyclone, United States), 1% penicillin/streptomycin (Tianjin Haoyang Biological Products Technology Co. Ltd., China), l-glutamine (Gibco, United States), and 10% fetal bovine serum (Gibco, United States). The iPSC-MSCs were cultured at 37°C in a 5% CO_2_ humidified incubator and the culture medium was changed every 2 days.

To prepare nanofiber scaffolds for cell culture, smooth glass sheets (diameter 15 mm) were cleaned, sterilized, and placed on an aluminum foil to collect the fibers. After electrospinning of the 2-ml solution, the prepared HAp/SF membranes were dried in a vacuum-drying chamber for at least 5 days to remove residual solvents. After the solvent was volatilized, the samples were placed in the corresponding cell-culture plate. Four-to-six samples for each structure were utilized, with blank cover slides placed into a plate as a control group. After 10 min of methanol treatment, the SF structure was transformed, methanol was removed, and sample scaffolds were irradiated by ultraviolet light for 2 days after methanol volatilization. The samples were soaked in 75% ethanol for 2 h, and then washed for 10 min with PBS (Phosphate Buffer Saline) three times. Each pore was added to the corresponding cell culture medium. The sample was soaked in an incubator overnight for preculture. The sample was prepared squarely and placed in a cell-culture plate. iPSC-MSCs were planted according to the experimental requirements.

### Effect of Scaffolds on Cell Morphology

Cell spreading on fibers was observed using SEM. To prepare the samples for SEM, the following procedure was used: 1) Cell–scaffold complexes were cultured for 1 and 7 days and then removed from the culture medium and washed with PBS three times; 2) 4% glutaraldehyde was added to the complexes for a period of 2.5 h or longer to immobilize the samples; 3) after the glutaraldehyde was removed, the complexes were washed by PBS three times; 4) samples were washed for 15 min in each of six concentrations of alcohol (10, 30, 50, 70, 90, and 100%) for dehydration; 4) treatment with hexamethyldisiloxane was carried out overnight. After drying, the structures were cut into fragments, sprayed with gold, and observed using SEM.

### Effects of Scaffolds on Cell Proliferation

The effects of the scaffolds on cell proliferation were evaluated by using cell counting kit-8 (CCK-8). At set times (1, 4, and 7 days), an appropriate volume of CCK-8 solution was added to the plates and incubated for 4 h. 200 μl of the culture medium was then extracted and added to a 96-well plate, without bubbles. The absorbance at 450 nm was determined using a microplate reader.

### Osteogenic Differentiation of iPSC-MSCs Induced by N-Acetyl Cysteine /HAp/SF Nanofibers *in vitro*

#### Quantitative Analysis of Alkaline Phosphatase (ALP)

A BCIP/NBT color development kit (Nanjing Jiancheng Bioengineering Institute, Nanjing, China) was used to assay ALP activity according to the manufacturer’s specifications. A quantitative analysis of ALP was performed using an ALP quantitative kit. The cell supernatant was removed and then 1% Triton X-100 (soluble in PBS) was added and incubated for 30 min to split the cells. 30 ml of the supernatant was distributed among a 96-well plate and then 50 μl of the buffer and matrix solution was added and incubated at 37°C for 15 min 150 μl of the chromogenic reagent was then added to the 96-well plate, shaken gently, and mixed evenly. The absorbance at 520 nm was determined using a microplate reader.

#### Quantitative Analysis of Collagen (COL)

Quantitative analysis of collagen was performed using a hydroxyproline testing kit (Nanjing Jiancheng Bioengineering Institute, Nanjing, China) as per the manufacturer’s instructions. In summary, the cell supernatant was removed and then 1% Triton X-100 (soluble in PBS) was added and incubated for 30 min to lyse the cells. The resulting solution, after lysis, was collected and pH was adjusted to 6.0–6.8. Reagent was added according to the hydroxyproline test kit and well mixed, followed by incubation at 60 °C in water for 15 min. After cooling and centrifugation for 10 min at 3,500 rpm, absorbance at 550 nm was measured.

### Detection of Osteogenesis-Related Genes

After 14 days incubation, the expressions of osteogenesis-related genes (ALP, COL, OCN (osteocalcin), and OPN (osteopontin)) in iPSC-MSCs cultured in HAp/SF, 10-NAC/HAp/SF, 20-NAC/HAp/SF nanofiber membranes, and the TCP blank control were detected by reverse transcription polymerase chain reaction (RT-PCR). All primer sequences were listed in Table 1. The procedure for RT-PCR included three principles processes: ribonucleic acid (RNA) extraction, reverse transcription deoxyribonucleic acid (DNA) synthesis, and RT-PCR gene amplification detection. RNA was rapidly extracted using a Biozol RNA extraction kit. Reverse transcription DNA synthesis was undertaken using a 20 L reactor containing 20–50 ng of RNA. RNA was reverse-transcribed to form complementary DNA (cDNA) using the FastQuant RT Kit (with gDNase). RT-PCR was performed using the SuperReal PreMix Plus (SYBR Green) kit, with the housekeeping gene (GAPDH) as a control. After 40 cycles, the relative expression of each target gene was calculated using the 2^−ΔΔct^ method.

### Bone Regeneration *in vivo*

#### Mouse Cranial Defect Model

After the *in vitro* osteogenesis study, we developed a mouse cranial defect model in 6-week-old male Sprague–Dawley (SD) rats (Shanghai Slac Laboratory Animal Co. Ltd., Shanghai, China) to analyze the efficacy of the nanofibrous HAp/SF scaffold in bone regeneration. Animal care and use protocols were implemented in accordance with the National Institutes of Health (NIH) Guide. The ethical committee of Yangzhou University approved the experimental procedures. Under general anesthesia, the cranium was exposed through a medial incision. The periosteum overlying the calvarial bone was completely resected. A dental bur was used to create a defect with a diameter of 3 mm per mouse. Nanofibrous SF, HAp/SF, NAC/SF, and NAC/HAp/SF scaffolds were implanted in the cranial defects, while the blank group was left untreated. Thirty mice (six in each set) were implanted. The skin was closed with a suture and the defects were analyzed 8 weeks after implantation.

### Computed Tomography (CT) and Bone Mineral Density Analysis

Dual-source CT (SOMATOM Definition, Siemens) was used to detect regeneration and quantify the mineral density of the newly formed bone within the cranial defects. Under general anesthesia, the mouse was fixed and scanned over the entire length at a voxel size of 12 l m and medium resolution of 80 kVp, 110 µA with a 0.5-mm Al filter. The regeneration of bone within the defects was visually identified and the manufacturer’s evaluation software was used to separate bone from non-bone and the mineral density of the new bone-like tissue was computed and calibrated to a HAp phantom.

### Statistical Analyses

All quantitative values are expressed as mean ± standard error of at least three replicate samples. An analysis of variance for all quantitative tests was carried out with the software Origin and Tukey’s honestly significant difference. *Post hoc* tests were used for pair-wise comparisons between groups. Statistical significance was defined by either *p < 0.05 or **p < 0.01.

## Results

### Morphology and Physical Properties

**FIGURE 1:**
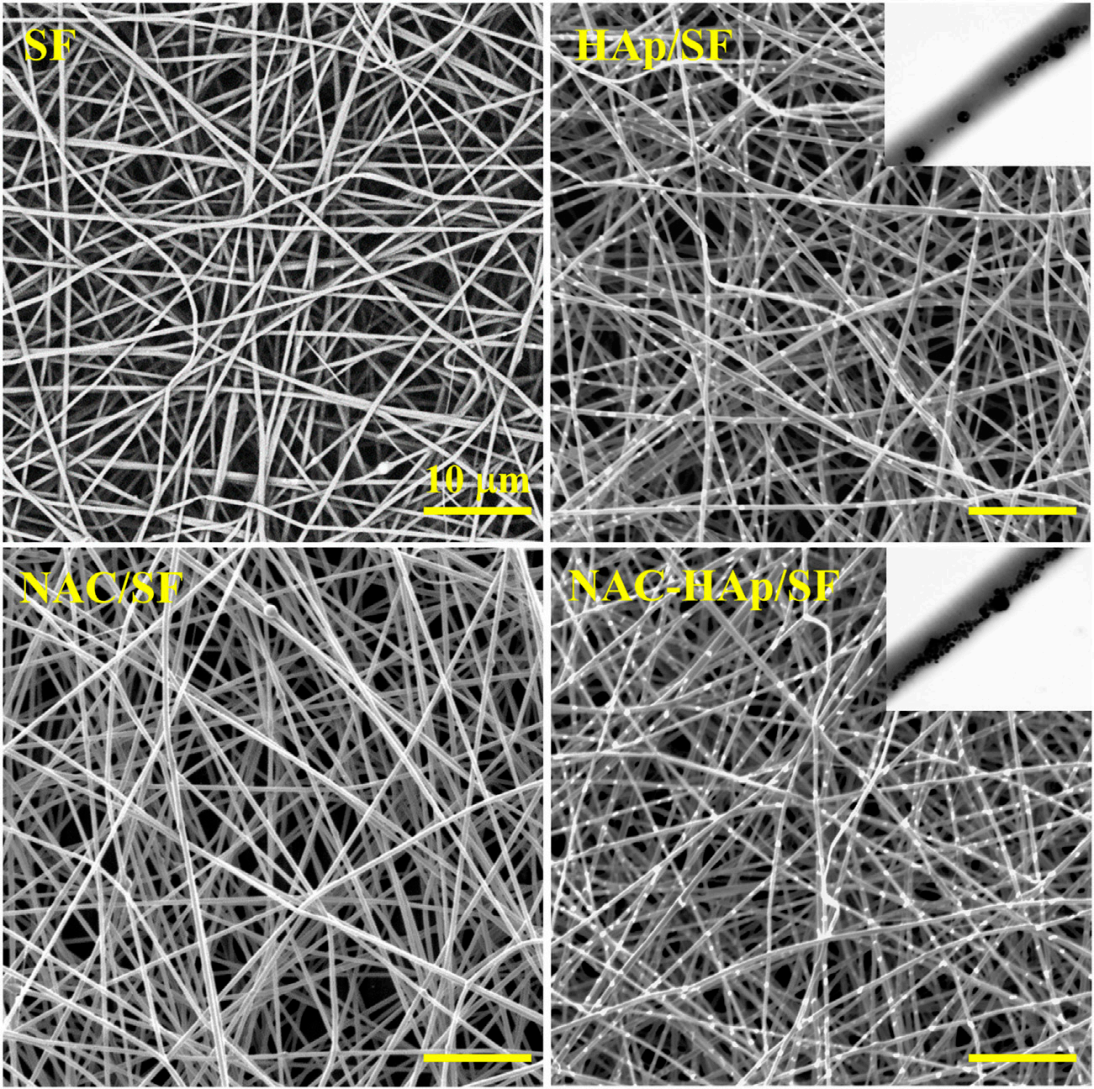
SEM images of the pure SF, HAp/SF, NAC/SF, and NAC/HAp/SF scaffolds; the insets are corresponding TEM images.

**FIGURE 2:**
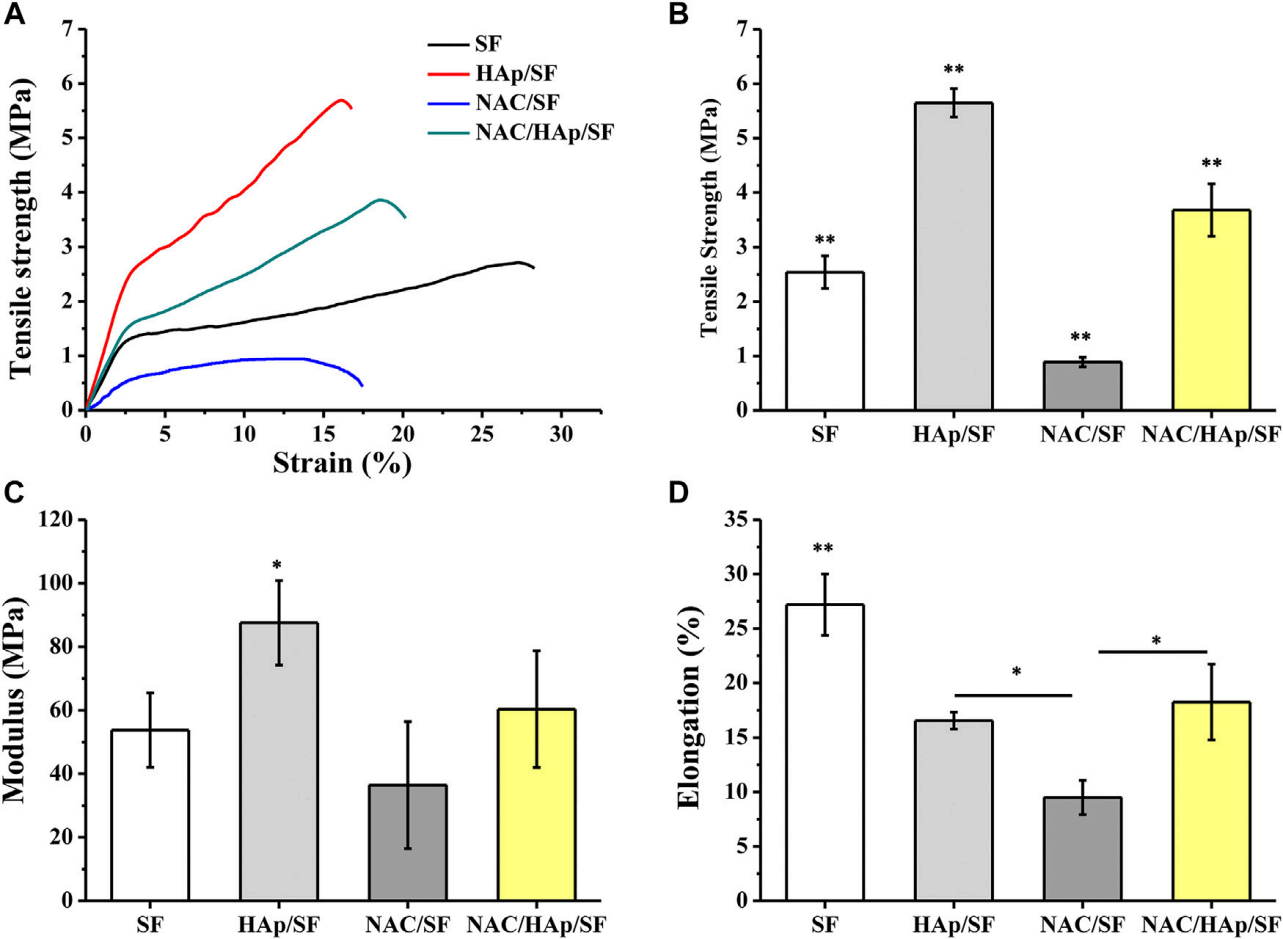
Analysis of tensile properties of the composite fibrous scaffolds including typical stress-strain curves **(A)**, tensile strength **(B)**, modulus **(C)** and elongation **(D)**. *p < 0.05, **p < 0.01, *n* = 5.

Composite fibers were prepared via electrospinning. The morphologies of the different electrospun fibers were observed by SEM (Figure 1) and all fibers exhibited a good morphology. White substances appeared on the surface of the fiber because of the aggregation of Hap. The TEM images showed that HAps were encapsulated within the electrospun silk fibers and the diameter of the fibers decreased with the incorporation of NAC and NAC/HAp, which may increase the conductivity and viscosity of the SF fibers, consistent with the results of Zhu et al. (2019). Typical tensile stress-strain curves and tensile mechanical properties of composite fiber films are shown in Figure 2. The addition of HAp, NAC and NAC/HAp had a significant effect on the mechanical properties of SF fibers. The average Young’s modulus and tensile strength of SF fibers were 53.76 ± 11.67 and 2.54 ± 0.30 MPa, respectively. With the addition of HAp, tensile strength and Young’s modulus significantly increased. The incorporation of NAC reduced the Young’s modulus and the tensile strength to 36.43 ± 19.99 MPa and 0.89 ± 0.09 MPa, respectively. When NAC/HAp were added, the Young’s modulus and the tensile strength increases significantly. The rate of elongation rate decreased with the addition of HAp, NAC and NAC/HAp into SF fibers. Generally speaking, the mechanical properties of the fibers were improved by adding NAC/HAp.

### Hydrophilic Properties of Fibers

**FIGURE 3:**
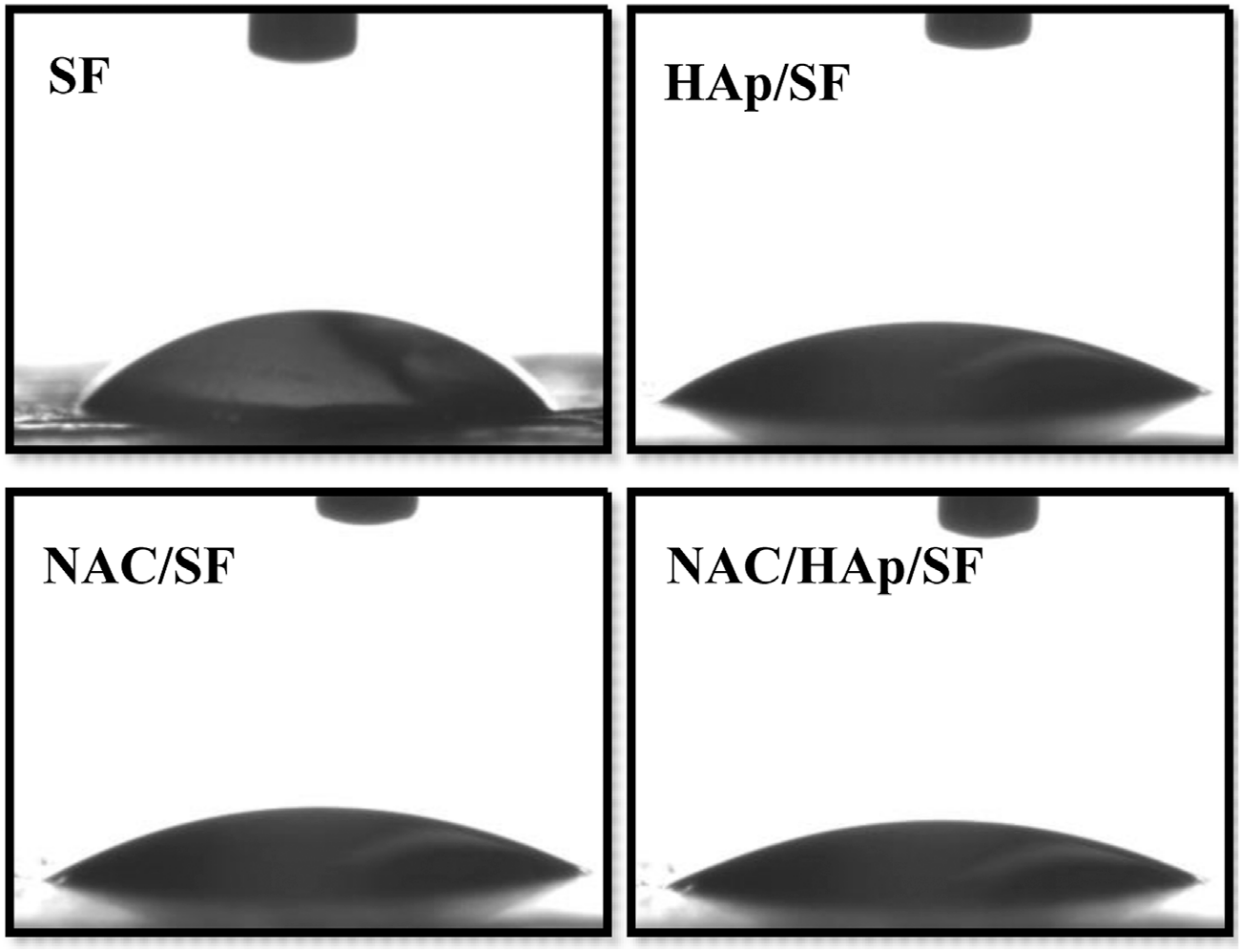
Hydrophilic properties of the SF, HAp/SF, NAC/SF, and NAC/HAp/SF composite fiber membranes, *n* = 3.

The hydrophilic angles of the composite fiber membranes were measured to evaluate their hydrophilic properties. Contact angles below 90° are indicative of hydrophilicity, which favors the diffusion of liquid on the materials. In contrast, contact angles exceeding 90° correspond to hydrophobicity (Trinca et al., 2017). The contact angles of NAC/HAp/SF were smaller than those of the other scaffolds (Figure 3). Thus the addition of NAC and HAps decreased the contact angle of the material surface through the synergy between NAC, which contains amine and carboxylic functional groups, and HAps, which contain hydroxyl groups.

### Drug Releasing Patterns of the Fiber-Loading Drug

**FIGURE 4:**
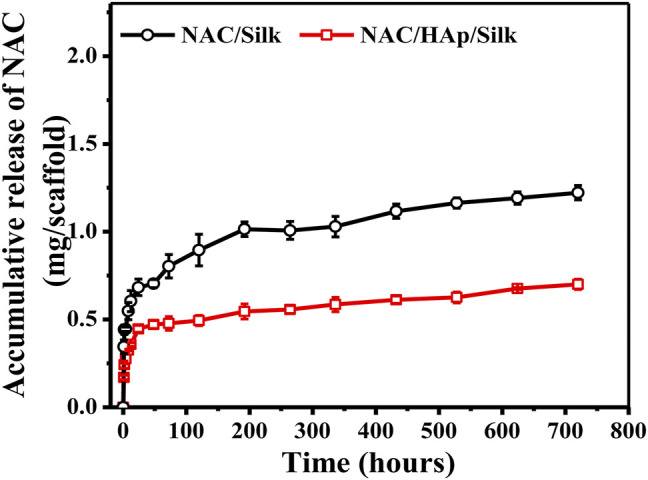
NAC release profiles of the NAC/SF and NAC/HAp/SF scaffolds during 0–720 h in a PBS solution, *n* = 3.

Figure 4 shows the cumulative release of NAC per Gram of the composite fiber scaffold. The NAC/SF group exhibited an initial burst of release; NAC then became exhausted after a period of time. In contrast, the release of NAC from the NAC/HAp/SF group exhibited a biphasic pattern, characterized by an initial burst of release followed by a long-term, gradual, and continuous release, during which high NAC concentrations were maintained sufficient to promote cell differentiation. In the first stage, the apparent fast release of NAC drugs may have occurred because some of the small NAC molecules dispersed on the surface of the fiber and the inner layer of the fiber *epidermis* during the electrospinning can be released quickly in the PBS buffer solution (Zheng et al., 2013). In the second stage, NAC inside the composite fiber was gradually released. This indicates that HAp exerted an adsorption effect on NAC and also a significant effect on the drug release behavior of NAC.

### Cell Proliferation

**FIGURE 5:**
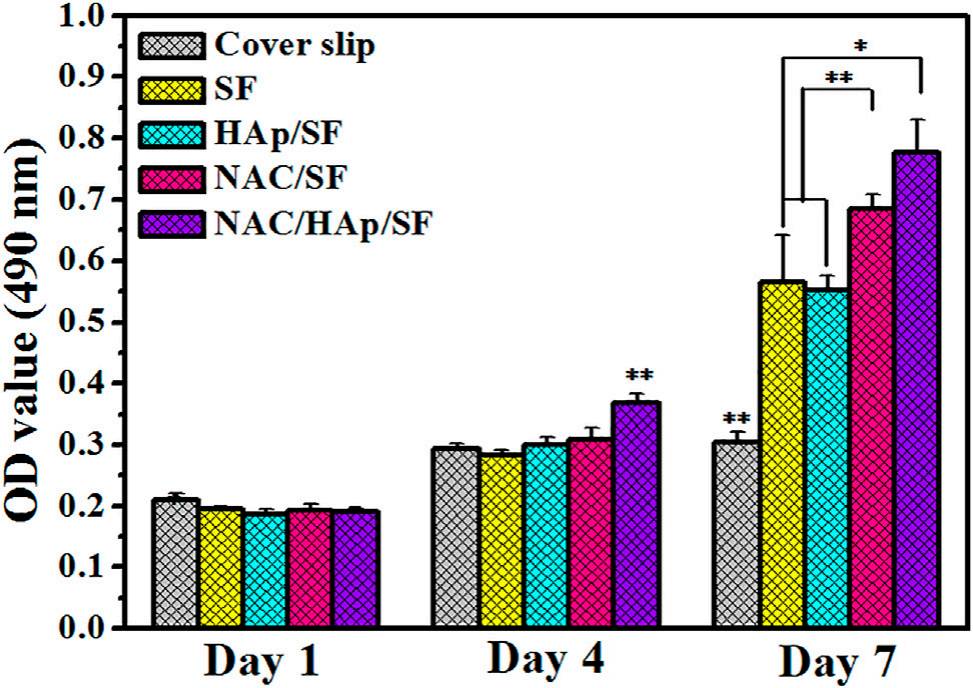
Proliferation of iPSC-MSCs on different composite fibers and cover slip, *n* = 3.

The CCK-8 assay was used to detect the proliferation of iPSC-MSCs cultured on composite fiber membranes (Figure 5). The cell content in all groups increased with the duration of incubation, which indicates that the cells were in a good state of proliferation. During the process of cell culture, the capacity for cell proliferation of all material groups was higher than that of the coverslip group. Cell proliferation activity was significantly higher in the NAC/SF and NAC/HAp/SF groups than in the control group. This suggests that the addition of NAC and HAps positively affected cell viability.

### Cell Morphology

**FIGURE 6:**
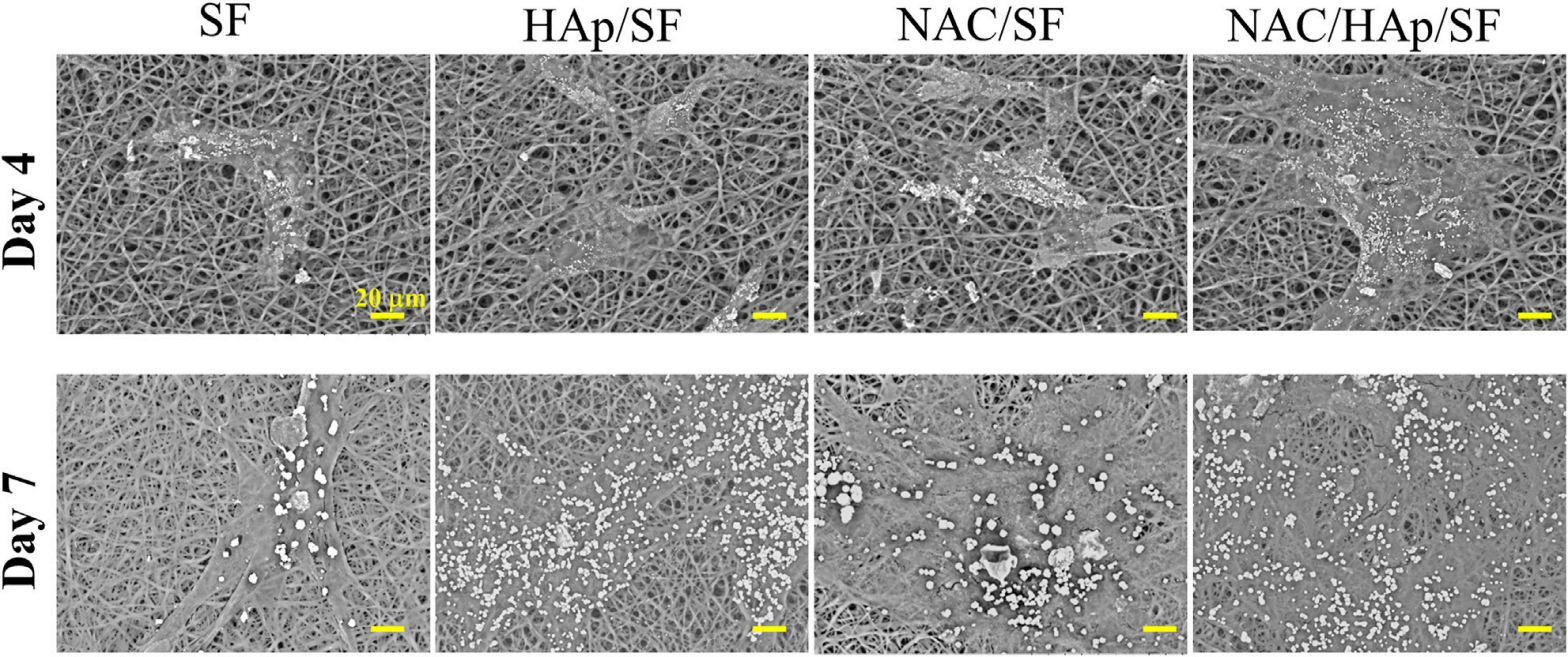
Morphological observation of iPSC-MSCs planted on different composite fibers for 4 and 7 days, *n* = 3.

The proliferation and spreading of iPSC-MSCs on different composite fibers were further observed by SEM (Figure 6). After 4 days of culture, the number of adherent cells in the NAC/HAp/SF group was higher than those in the other groups. After 7 days of culture, the number of cells on the fiber increased and the spread of the cells were incorporated with the fiber scaffold. From the perspective of the number of fiber cells, this trend was consistent with the CCK8 results.

### Expression of Osteoblast-Related Genes in Cells

**FIGURE 7:**
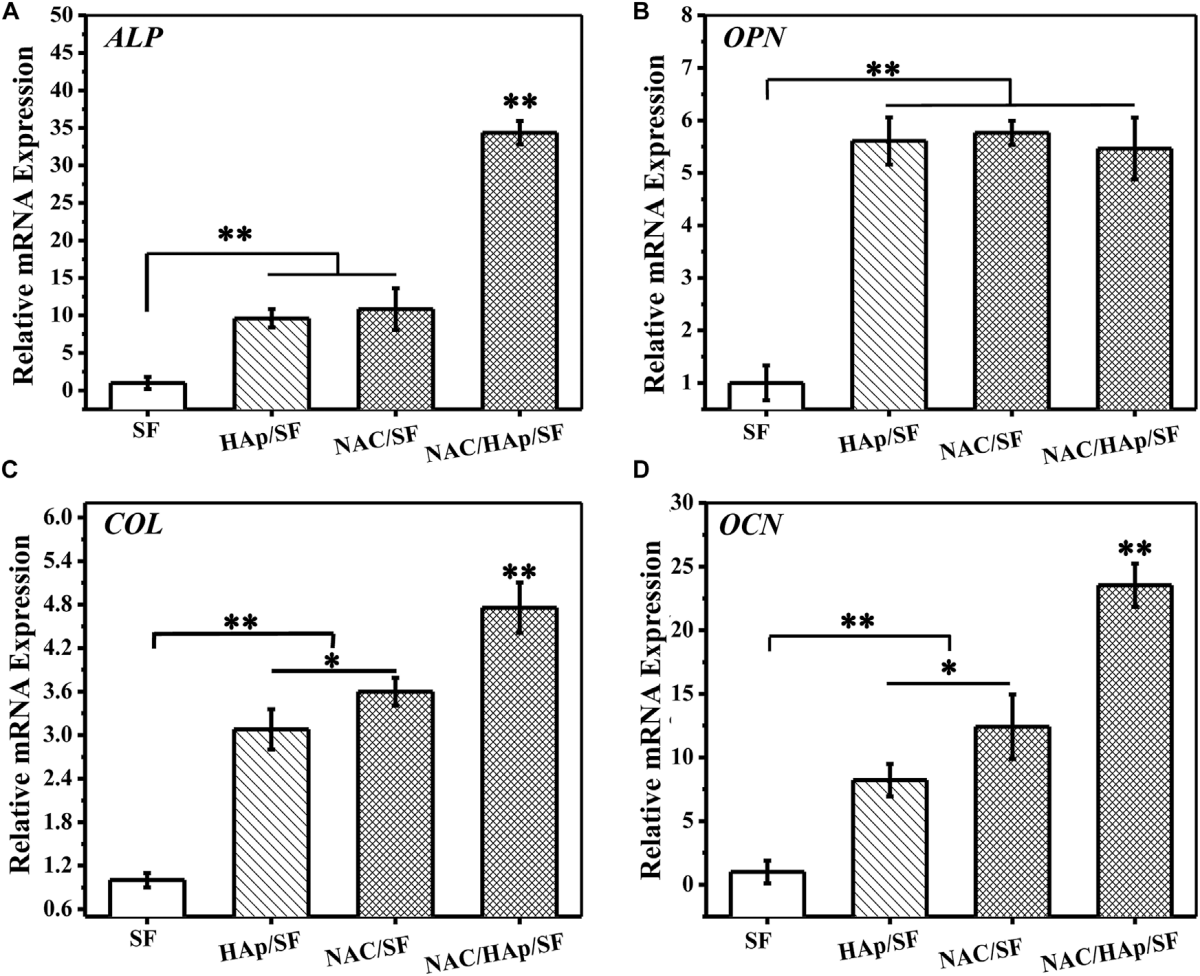
Expressions of bone-related genes in iPSC-MSCs on different nanofibers for 14 days, *n* = 3.

To evaluate the capacity for osteogenic differentiation of iPSC-MSCs on different nanofiber scaffolds, the marker genes of osteogenic differentiation (*ALP*, *COL*, *OCN*, and *OPN*) were detected by RT-PCR. After 14 days of culture, cells in the corresponding culture plate were collected and RNA was extracted for detection. The SF group was used as a control group in this experiment. The expression of *ALP* in the NAC/HAp/SF fibers increased significantly after 14 days (Figure 7). This may be explained by the fact that as the fibers promote the osteogenic differentiation of iPSC-MSCs and the cell differentiation process is in the middle and late stages, the content of the early expression product of *ALP* gradually decreases. With the addition of HAp and NAC, the expression levels of *COL* and *OCN* were significantly increased (*p* < 0.01). According to the gene expression level of *OPN*, the addition of HAp increased the level of *OPN* after 14 days of cell culture, while the addition of NAC did not affect the expression of *OPN*. The addition of NAC increased not only the level of expression of *ALP*, but also those of *COL* and *OCN*, the marker genes in the middle and late stages of osteogenesis but had no significant effect on the expression of *OPN*. However, compared to the control group, HAp and NAC complemented each other and promoted the expression of *ALP*, *COL*, *OCN*, and *OPN* genes, thus promoting the osteogenic differentiation of iPSC-MSCs.

### Expression of ALP in Cells

**FIGURE 8:**
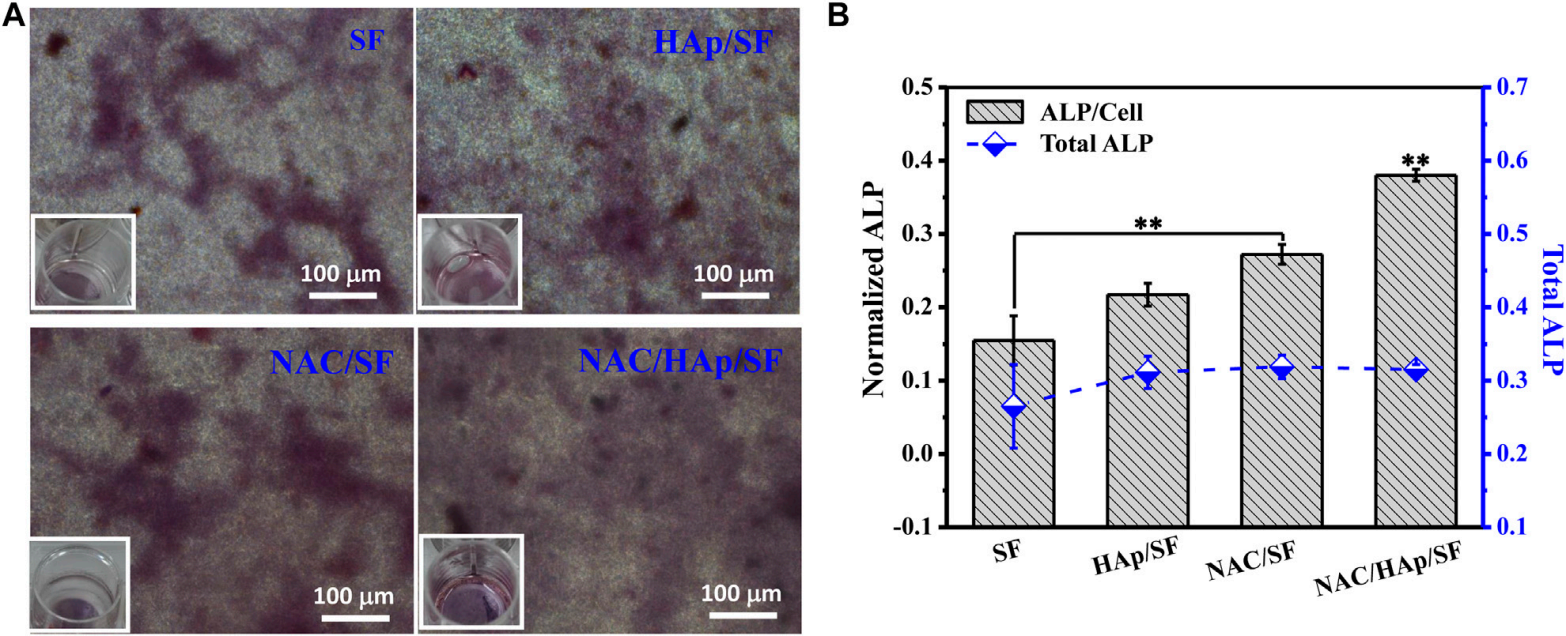
ALP staining. **(A)** and quantitative detection of ALP. **(B)** after iPSC-MSC culture on different composite fibers for 14 days, *n* = 3.

iPSC-MSCs were planted on different composite fibers. After 14 days of culture, ALP staining and quantitative analysis of ALP secreted by cells, including the total amount of ALP and amount of ALP secreted by a single iPSC-MSC, were undertaken. The results are shown in Figure 8. The addition of HAp resulted in an increase in the expression of ALP in iPSC-MSCs. The addition of NAC also promoted the expression of ALP in iPSC-MSCs, which indicated that it significantly enhanced the osteogenic differentiation of iPSC-MSCs.

### Expression of COL in Cells

**FIGURE 9:**
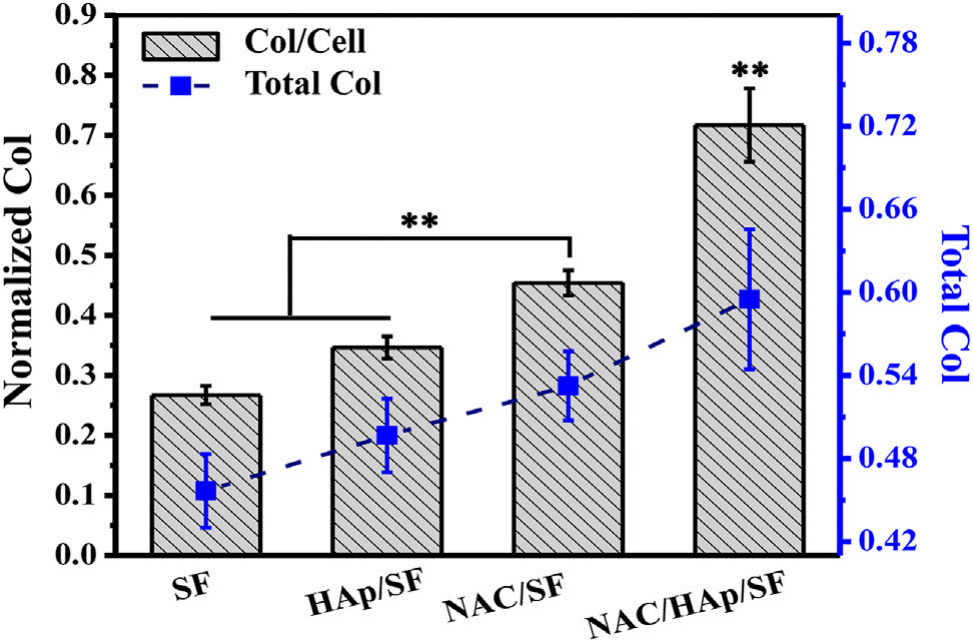
Quantitative results of COL after 14 days of culture of iPSC-MSCs on different nanofibers, *n* = 3.

iPSC-MSCs were seeded on composite fibers. After 14 days of culture, collagen was quantitatively examined using a testing kit (Figure 9). The detection results were similar to the results of cellular ALP expression. that is, the loading of NAC promoted not only the expression of total cell COL, but also the expression of COL in a single cell. The total COL expression of all cells and expression of COL in single cells on NAC/HAp/SF fibers were significantly higher than those in the other groups. This indicates that the NAC/HAp/SF composite nanofibers had the highest ability to promote collagen production. Thus, the loading of NAC can effectively improve the osteogenic differentiation performance of the fibers.

### *In-vivo* Bone Regeneration

**FIGURE 10:**
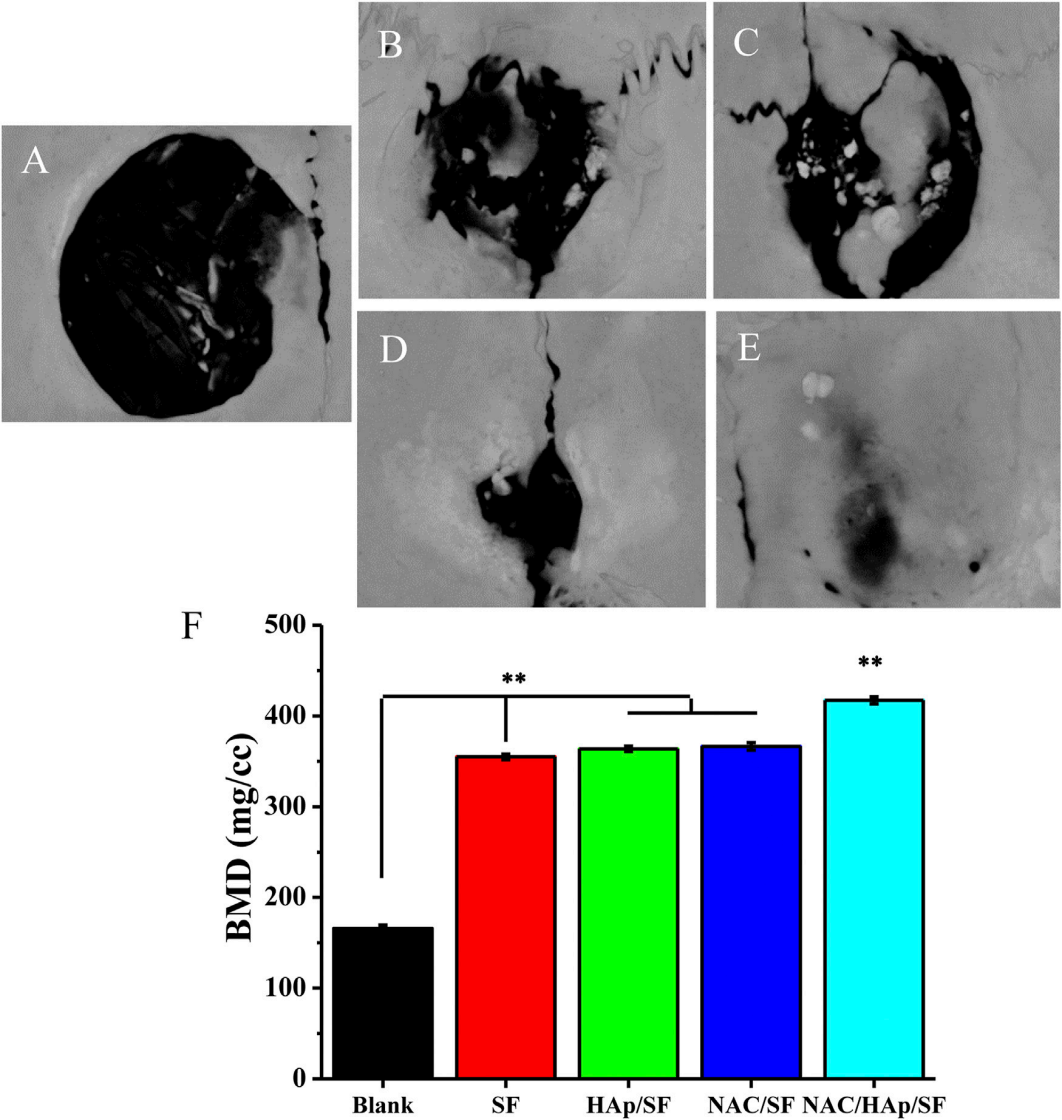
CT examination of the whole calvarias **(A–E)** and bone mineral density of the defects **(F)** 8 weeks after implantation *in vivo* **(A)** blank, **(B)** SF, **(C)** HAp/SF, **(D)** NAC/SF, and **(E)** NAC/HAp/SF groups. *p < 0.05, **p < 0.01, *n* = 3.

To verify the capability of the biomimetic scaffold of NAC/HAp/SF to regenerate bone *in vivo*, SF, HAp/SF, NAC/SF, and NAC/HAp/SF scaffolds were transplanted into calvarial defects in SD rats. After 8 weeks of implantation, CT images demonstrated that the whole defect was almost fully repaired by the formation of bone-like tissues in the NAC/HAp/SF group (Figure 10). Moreover, the bone mineral density was significantly increased (*p* < 0.01), peaking at 2.51, 1.18, 1.15, and 1.14 times those of the blank SF, HAp/SF, NAC/SF, and NAC/HAp/SF groups, respectively (Figure 10F). Notably, the NAC/HAp/SF scaffold without cells exhibited the highest capacity for repair (Figures 10A–D) and highest bone mineral density compared to the other groups (Figure 10F). The bone mineral density was higher in the NAC/SF group than in the HAp/SF group, but not significantly. The NAC/HAp/SF led to the best outcomes in regenerating cranial bone defects in rats.

## Discussion

Massive bone defects, defined as critical-size bone defects, can cause delayed union and non-union, and even limb dysfunction. There are several origins of massive bone defects, including trauma, bone tumor resection, and revision arthroplasty (Yamada et al., 2013; Yang et al., 2014).

Numerous small molecules regulate the differentiation and proliferation of various cells (Xu et al., 2008). NAC is a water-soluble compound with a low molecular weight. It has antioxidant properties and enhances cytocompatibility (Zafarullah et al., 2003; Watanabe et al., 2018). NAC loaded on a collagenous sponge scaffold can promote bone regeneration by accelerating osteogenesis (Yamada et al., 2013).

SF is a natural biopolymer and promising drug carrier owing to its high biocompatibility, tailorable biodegradability, and low bacterial attachment. SF-based scaffolds have been considered potential carriers for drug delivery (Farokhi et al., 2016; Farokhi et al., 2019; Farokhi et al., 2020). HAp is widely and clinically used to generate bone tissue because of its high-efficiency osteogenesis (He et al., 2012). Scaffolds have an important role in bone tissue generation, particularly electrospun scaffolds, which have good characteristics for applications in drug delivery and bone repair (Xue et al., 2017; Ko et al., 2018).

In this study, we developed an HAp/SF scaffold through electrospinning and NAC was loaded onto the HAP/SF scaffold. We analyzed the optimal amount of NAC in the preparation of fibers and evaluated its physical properties, including the morphology, mechanical properties, and hydrophilic properties. The release of NAC in the NAC/HAp/SF compound scaffold exhibited a biphasic pattern characterized by an initial burst in release followed by a long-term, gradual, and continuous release stage to promote cell differentiation due to high NAC concentrations. In addition, HAp was beneficial to the sustained release of the drug.

The cell compatibility of the NAC/HAp/SF composite fibers was then evaluated. All drug-loaded fibers were beneficial for cell adhesion, spreading, and proliferation. We detected osteogenic differentiation of iPSC-MSCs. At the protein level (ALP and COL) or gene level (*ALP*, *COL*, *OCN*, and *OPN*), the addition of NAC significantly promoted osteogenic differentiation of iPSC-MSCs. NAC and HAp complemented each other and cooperated to promote the expression of *ALP*, *COL*, *OCN*, and *OPN* genes. The NAC/HAp/SF fiber scaffolds exhibited good biocompatibility and safety and induced osteogenic differentiation of iPSC-MSCs.

To further investigate the capacity for osteogenesis of the NAC/HAp/SF scaffold, we implemented cranial bone defects in rats. After 8 weeks, CT imaging confirmed the capacity of the NAC/HAp/SF scaffolds to repair cranial bone defects. The electrospun nanofibrous NAC/HAp/SF scaffold, with an excellent osteo-induction effect, may be ideal for the regulation of osteogenic differentiation of iPSC-MSCs for patient-specific repair and regeneration of bone tissue in the future.

## Conclusion

In this study, we formed NAC/HAp/SF scaffolds by electrospinning and analyzed the release of NAC and their ability to promote cell proliferation and osteogenesis. In addition, we detected osteogenesis in NAC/HAp/SF scaffolds in rats. Although satisfactory results were obtained, shortcomings remain. For example, the period of repair of 8 weeks in the rat body is short. Furthermore, the exact mechanism of the effect of the NAC/HAp/SF scaffolds on bone generation is unclear. An extended period of observation and evaluation of the exact mechanism of osteogenesis of the NAC/HAp/SF scaffolds should be performed in further studies.

**TABLE 1:** Primer sequences of bone-related genes.

Genes	Forward primer sequence (5′-3′)	Reverse primer sequence (5′-3′)
OCN	CAG​TAA​GGT​GGT​GAA​TAG​ACT​CCG	GGT​GCC​ATA​GAT​GCG​CTT​G
COL	GGT​CCC​AAA​GGT​GCT​GAT​GG	GAC​CAG​GCT​CAC​CAC​GGT​CT
ALP	GTC​CCA​CAA​GAG​CCC​ACA​AT	CAA​CGG​CAG​AGC​CAG​GAA​T
OPN	GCT​TCC​TGC​TCA​TCA​ATC​GTA​AC	TCA​TCT​GCC​GAC​CCT​CTT​CT
GAPDH	GGC​AAG​TTC​AAC​GGC​ACA​GT	GCC​AGT​AGA​CTC​CAC​GAC​AT

## Data Availability

The original contributions presented in the study are included in the article/Supplementary Material, further inquiries can be directed to the corresponding authors

## References

[B1] Banaszynski L. A. Chen L.-c. Maynard-Smith L. A. Ooi A. G. L. Wandless T. J. (2006). A Rapid, Reversible, and Tunable Method to Regulate Protein Function in Living Cells Using Synthetic Small Molecules. Cell 126, 995–1004. 10.1016/j.cell.2006.07.025 16959577 PMC3290523

[B2] Boccaccini A. R. Blaker J. J. (2005). Bioactive Composite Materials for Tissue Engineering Scaffolds. Expert Rev. Med. Devices 2, 303–317. 10.1586/17434440.2.3.303 16288594

[B3] Collignon A.-M. Lesieur J. Vacher C. Chaussain C. Rochefort G. Y. (2017). Strategies Developed to Induce, Direct, and Potentiate Bone Healing. Front. Physiol. 8, 927. 10.3389/fphys.2017.00927 29184512 PMC5694432

[B4] Du X. Xie Y. Xian C. J. Chen L. (2012). Role of FGFs/FGFRs in Skeletal Development and Bone Regeneration. J. Cel. Physiol. 227, 3731–3743. 10.1002/jcp.24083 22378383

[B5] Farokhi M. Mottaghitalab F. Fatahi Y. Saeb M. R. Zarrintaj P. Kundu S. C. (2019). Silk Fibroin Scaffolds for Common Cartilage Injuries: Possibilities for Future Clinical Applications. Eur. Polym. J. 115, 251–267. 10.1016/j.eurpolymj.2019.03.035

[B6] Farokhi M. Mottaghitalab F. Reis R. L. Ramakrishna S. Kundu S. C. (2020). Functionalized Silk Fibroin Nanofibers as Drug Carriers: Advantages and Challenges. J. Controlled Release 321, 324–347. 10.1016/j.jconrel.2020.02.022 32061791

[B7] Farokhi M. Mottaghitalab F. Samani S. Shokrgozar M. A. Kundu S. C. Reis R. L. (2018). Silk Fibroin/hydroxyapatite Composites for Bone Tissue Engineering. Biotechnol. Adv. 36, 68–91. 10.1016/j.biotechadv.2017.10.001 28993220

[B8] Farokhi M. Mottaghitalab F. Shokrgozar M. A. Kaplan D. L. Kim H.-W. Kundu S. C. (2016). Prospects of Peripheral Nerve Tissue Engineering Using Nerve Guide Conduits Based on Silk Fibroin Protein and Other Biopolymers. Int. Mater. Rev. 62, 367–391. 10.1080/09506608.2016.1252551

[B9] Groeneveld E. Burger E. (2000). Bone Morphogenetic Proteins in Human Bone Regeneration. Eur. J. Endocrinol. 142, 9–21. 10.1530/eje.0.1420009 10633215

[B10] He J. Wang D. Cui S. (2012). Novel Hydroxyapatite/tussah Silk Fibroin/chitosan Bone-like Nanocomposites. Polym. Bull. 68, 1765–1776. 10.1007/s00289-012-0702-5

[B11] Hong L. Tabata Y. Miyamoto S. Yamada K. Aoyama I. Tamura M. (2000). Promoted Bone Healing at a Rabbit Skull Gap between Autologous Bone Fragment and the Surrounding Intact Bone with Biodegradable Microspheres Containing Transforming Growth Factor-Β1. Tissue Eng. 6, 331–340. 10.1089/107632700418056 10992430

[B12] James A. W. Lachaud G. Shen J. Asatrian G. Nguyen V. Zhang X. (2016). A Review of the Clinical Side Effects of Bone Morphogenetic Protein-2. Tissue Eng. B: Rev. 22, 284–297. 10.1089/ten.teb.2015.0357 PMC4964756 26857241

[B13] Ko E. Lee J. S. Kim H. Yang S. Y. Yang D. Yang K. (2018). Electrospun Silk Fibroin Nanofibrous Scaffolds with Two-Stage Hydroxyapatite Functionalization for Enhancing the Osteogenic Differentiation of Human Adipose-Derived Mesenchymal Stem Cells. ACS Appl. Mater. Inter. 10, 7614–7625. 10.1021/acsami.7b03328 28475306

[B14] Koch H. Jadlowiec J. A. Campbell P. G. (2005). Insulin-like Growth Factor-I Induces Early Osteoblast Gene Expression in Human Mesenchymal Stem Cells. Stem Cell Dev. 14, 621–631. 10.1089/scd.2005.14.621 16433617

[B15] Mayr-Wohlfart U. Waltenberger J. Hausser H. Kessler S. Günther K.-P. Dehio C. (2002). Vascular Endothelial Growth Factor Stimulates Chemotactic Migration of Primary Human Osteoblasts. Bone 30, 472–477. 10.1016/s8756-3282(01)00690-1 11882460

[B16] Oikawa S. Yamada K. Yamashita N. Tada-Oikawa S. Kawanishi S. (1999). N-acetylcysteine, a Cancer Chemopreventive Agent, Causes Oxidative Damage to Cellular and Isolated DNA. Carcinogenesis 20, 1485–1490. 10.1093/carcin/20.8.1485 10426796

[B17] Ontoria J. M. Altamura S. Di Marco A. Ferrigno F. Laufer R. Muraglia E. (2009). Identification of Novel, Selective, and Stable Inhibitors of Class II Histone Deacetylases. Validation Studies of the Inhibition of the Enzymatic Activity of HDAC4 by Small Molecules as a Novel Approach for Cancer Therapy. J. Med. Chem. 52, 6782–6789. 10.1021/jm900555u 19888759

[B18] Sell S. Barnes C. Smith M. Mcclure M. Madurantakam P. Grant J. (2007). Extracellular Matrix Regenerated: Tissue Engineering via Electrospun Biomimetic Nanofibers. Polym. Int. 56, 1349–1360. 10.1002/pi.2344

[B19] Trinca R. B. Westin C. B. Da Silva J. A. F. Moraes Â. M. (2017). Electrospun Multilayer Chitosan Scaffolds as Potential Wound Dressings for Skin Lesions. Eur. Polym. J. 88, 161–170. 10.1016/j.eurpolymj.2017.01.021

[B20] Watanabe J. Yamada M. Niibe K. Zhang M. Kondo T. Ishibashi M. (2018). Preconditioning of Bone Marrow-Derived Mesenchymal Stem Cells with N-Acetyl-L-Cysteine Enhances Bone Regeneration via Reinforced Resistance to Oxidative Stress. Biomaterials 185, 25–38. 10.1016/j.biomaterials.2018.08.055 30216807

[B21] Xu Y. Shi Y. Ding S. (2008). A Chemical Approach to Stem-Cell Biology and Regenerative Medicine. Nature 453, 338–344. 10.1038/nature07042 18480815

[B22] Xue J. Xie J. Liu W. Xia Y. (2017). Electrospun Nanofibers: New Concepts, Materials, and Applications. Acc. Chem. Res. 50, 1976–1987. 10.1021/acs.accounts.7b00218 28777535 PMC6589094

[B23] Yamada M. Tsukimura N. Ikeda T. Sugita Y. Att W. Kojima N. (2013). N-acetyl Cysteine as an Osteogenesis-Enhancing Molecule for Bone Regeneration. Biomaterials 34, 6147–6156. 10.1016/j.biomaterials.2013.04.064 23711675

[B24] Yang X. Li E. Wan Y. Smith P. Shang G. Cui Q. (2014). Antioxidative Fullerol Promotes Osteogenesis of Human Adipose-Derived Stem Cells. Int. J. Nanomedicine. 9, 4023–4031. 10.2147/ijn.s66785 25187705 PMC4149442

[B25] Zafarullah M. Li W. Q. Sylvester J. Ahmad M. (2003). Molecular Mechanisms of N -acetylcysteine Actions. Cell Mol. Life Sci. (Cmls) 60, 6–20. 10.1007/s000180300001 12613655 PMC11138873

[B26] Zheng F. Wang S. Wen S. Shen M. Zhu M. Shi X. (2013). Characterization and Antibacterial Activity of Amoxicillin-Loaded Electrospun Nano-Hydroxyapatite/poly(lactic-Co-Glycolic Acid) Composite Nanofibers. Biomaterials 34, 1402–1412. 10.1016/j.biomaterials.2012.10.071 23168384

[B27] Zhu Y. Gu Y.-x. Mo J.-j. Shi J.-y. Qiao S.-c. Lai H.-c. (2015). N-acetyl Cysteine Protects Human Oral Keratinocytes from Bis-GMA-Induced Apoptosis and Cell Cycle Arrest by Inhibiting Reactive Oxygen Species-Mediated Mitochondrial Dysfunction and the PI3K/Akt Pathway. Toxicol. Vitro 29, 2089–2101. 10.1016/j.tiv.2015.09.002 26343756

[B28] Zhu Y. Song F. Ju Y. Huang L. Zhang L. Tang C. (2019). NAC-loaded Electrospun Scaffolding System with Dual Compartments for the Osteogenesis of rBMSCs *In Vitro* . Nt J. Nanomedicine 14, 787–798. 10.2147/ijn.s183233 PMC6361317 30774333

